# NOON-state interference in the frequency domain

**DOI:** 10.1038/s41377-024-01439-9

**Published:** 2024-04-15

**Authors:** Dongjin Lee, Woncheol Shin, Sebae Park, Junyeop Kim, Heedeuk Shin

**Affiliations:** Department of Physics, Pohang University of Science and Technology (POSTECH), Pohang, 37673 South Korea

**Keywords:** Quantum optics, Single photons and quantum effects, Nonlinear optics

## Abstract

The examination of entanglement across various degrees of freedom has been pivotal in augmenting our understanding of fundamental physics, extending to high dimensional quantum states, and promising the scalability of quantum technologies. In this paper, we demonstrate the photon number path entanglement in the frequency domain by implementing a frequency beam splitter that converts the single-photon frequency to another with 50% probability using Bragg scattering four-wave mixing. The two-photon NOON state in a single-mode fiber is generated in the frequency domain, manifesting the two-photon interference with two-fold enhanced resolution compared to that of single-photon interference, showing the outstanding stability of the interferometer. This successful translation of quantum states in the frequency domain will pave the way toward the discovery of fascinating quantum phenomena and scalable quantum information processing.

## Abstract (web summary)

We report the NOON-state interference in the frequency domain. Two photons with distinct colors, blue and red, are injected into an interferometer constructed with two frequency beam splitters. The resultant interference pattern is then observed.

## Graphical abstract

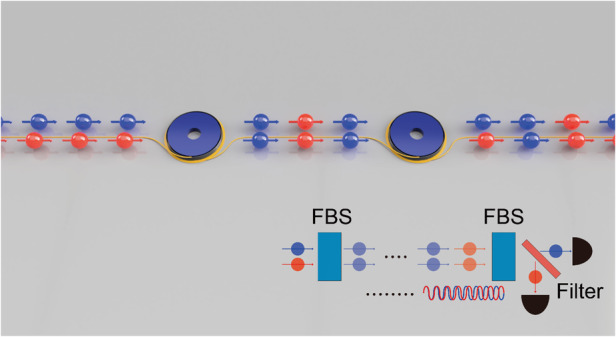

## Introduction

Photonic entanglement plays a crucial role in resolving fundamental questions in quantum mechanics and exploiting quantum information technology’s merits^1^. Extensive investigations have been conducted into entanglement in various degrees of freedom, including polarization^2^, path^3^, orbital angular momentum^4^, and time-bin^5^. These studies have been key in investigating diverse quantum phenomena, enhancing the performance of quantum communications^6,7^, protecting quantum states from environment noise^8^, and surpassing classical limitations in metrology^9–12^.

The NOON state, denoted as $\left|{\rm{\psi }}\right\rangle ={\left({\rm{|N}}\right\rangle }_{1}{\left|0\right\rangle }_{2}+{\left|0\right\rangle }_{1}{\left|{\rm{N}}\right\rangle }_{2})/\sqrt{2}$, is commonly referred to as the photon number path entangled state. This state represents a superposition between *N* photons in path 1 and zero photons in path 2 and vice-versa. A distinctive feature of the NOON state is its *N*-fold enhancement in phase sensitivity, which allows it to surpass the limitations of classical light measurement^9,13^. This attribute has made the NOON state a fundamental resource in quantum applications, including quantum lithography^10^, quantum imaging^14^, and quantum metrology^11,15^. Moreover, the unique entanglement properties of the NOON state pave the way toward the exploration of diverse topics in quantum information science, such as nonlocality^16^, quantum error correction^17^, and tight-binding model^18,19^.

Recently, the focus on quantum states in the frequency domain has intensified due to its potential for high-dimensional state extensibility, spatial single-mode propagation, stability, miniaturization, and compatibility with fiber networks^20–24^. The potential benefits have spurred extensive research into a variety of techniques for photon creation and manipulation within the frequency domain^20,22–26^. Furthermore, frequency-domain classical light controls have facilitated the demonstration of complex physical phenomena, including three-dimensional photonic topological insulator^27^ and complex long-range coupling^28,29^.

In this work, we demonstrate the NOON-state interference in the frequency domain for the first time, which is a crucial resource in quantum optics, to the best of our knowledge. A quantum frequency translation process acts like a 50:50 beam splitter with about a 50% probability of converting a single-photon frequency to another and enables the creation of the *N* = 2 NOON state in the frequency domain using a nondegenerate photon pair. The state is subsequently reintroduced into the frequency beam splitter through reflection, and the relative phase of the NOON state is controlled via a variable delay line. We observe two-photon bunching and anti-bunching effects in the frequency domain against the relative phase, with the oscillation frequency of the NOON state displaying a twofold enhancement compared to single-photon interference. Furthermore, our approach ensures an extremely stable interferometer due to the single-mode propagation of two-color components, even without any stabilization method. Consequently, our work represents a significant step toward exploring novel quantum effects and facilitating new tools for quantum information processing.

## Results

**Fig. 1:**
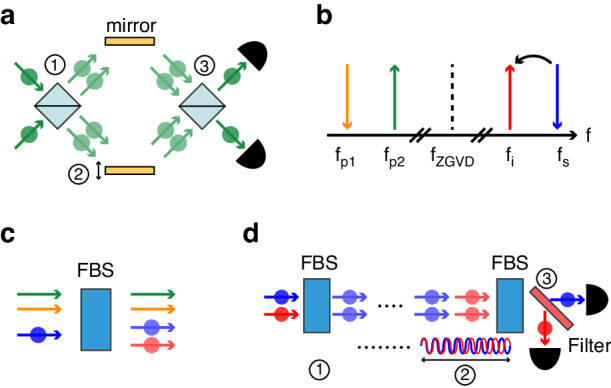
**Schematic diagram. a** Traditional NOON-state interference (*N* = 2) scheme in the spatial domain. **b** BS-FWM frequency diagram. *f*_xx_ (${\rm{xx}}={\rm{p}}1,{\rm{p}}2,{\rm{s}},{\rm{i}},{\rm{ZGVD}}$) indicates the frequencies of two pump fields, signal photon, idler photon, and the zero-group-velocity dispersion (ZGVD) of a nonlinear medium, respectively. **c** Schematic diagram of a frequency beam splitter through the BS-FWM process with two pump fields, an input photon, and a target photon. FBS frequency beam splitter. **d** Proposed NOON-state interference scheme in the frequency domain

Figure 1 illustrates our experimental diagram. The traditional NOON-state interference in the spatial domain is shown in Fig. 1a. Here, ① two indistinguishable single photons are simultaneously introduced into two input modes of a beam splitter, yielding the Hong-Ou-Mandel (HOM) effect and creation of a NOON state (*N* = 2). ② We can control the relative phase between the two paths by sliding a mirror, and ③ the NOON state can be combined by the second beam splitter. The resultant state exhibits two-photon bunching and anti-bunching effects in the path modes, contingent on the relative phase. The bunching cycles of the NOON state (*N* = 2) display a twofold enhancement compared to single-photon interference.

In this study, we generate the NOON state in the frequency domain using optical elements that serve functions analogous to those in traditional methods. We utilize frequency beam splitters based on quantum frequency translation, akin to the spatial beam splitters. The frequency translation method used in this study is BS-FWM^20,21,30^, which facilitates the simultaneous annihilation of an input photon and the creation of a target photon, driven by two classical pump fields^31,32^, as seen in Fig. 1b. From now on, we denote the input and target modes as signal and idler modes, respectively. The idler photon’s frequency obeys the energy conservation and phase-matching conditions, which can be fulfilled by symmetrically placing the four fields around the ZGVD frequency of the nonlinear material. The idler photon’s frequency is determined by ${{f}}_{{i}}={{f}}_{{\rm{s}}}+{{f}}_{{\rm{p}}1}-{{f}}_{{\rm{p}}2}$, where *f*_l_ ($\tt {l}={i},{s},{p}1,{p}2$) represents the frequency of the idler, signal, pump1, and pump2, respectively. Vertical arrows pointing upwards signify the creation process, while those pointing downwards represent the annihilation process. The evolution of the annihilation mode operators via BS-FWM is dictated by^21,31,32^

1 $${\hat{{a}}}_{{\rm{s}},{\rm{out}}}=\cos \left({\rm{gL}}\right){\hat{{{a}}}}_{{\rm{s}},{\rm{in}}}+{{\rm{ie}}}^{{i}{\rm{\phi }}}\sin ({\rm{gL}}){\hat{{{a}}}}_{{\rm{i}},{\rm{in}}}$$

2 $${\hat{{{a}}}}_{{\rm{i}},{\rm{out}}}={{\rm{ie}}}^{-{{i}}{\rm{\phi }}}\sin ({\rm{gL}}){\hat{{{a}}}}_{{\rm{s}},{\rm{in}}}+\cos ({\rm{gL}}){\hat{{{a}}}}_{{\rm{i}},{\rm{in}}}$$

where ${\hat{{\rm{a}}}}_{{\rm{m}},{\rm{n}}}$ ($\tt {{m}}={{s}},{{i;n}}={\rm{in}},{\rm{out}}$) denotes the annihilation operators of signal and idler modes in the input and output modes, respectively. *L* symbolizes the length of the nonlinear medium, and ϕ signifies the phase difference between the two pump beams. The parameter *g* is defined as ${{g}}\equiv {{\gamma }}{{P}}$, where *γ* is the nonlinear coefficient and *P*_1_ and *P*_2_ are the powers of the pump beam 1 and 2, respectively. In this study, *P* is the total power of the two pump beams, which are set to be equal (${{P}}/2={{{P}}}_{1}={{{P}}}_{2}$). This ensures the attainment of the phase-matching condition, independent of the total pump power^31^. Eqs. (1) and (2) equate to operators describing the functionality of a frequency beam splitter. A frequency beam splitter allows frequency translation of a signal photon to the desired idler frequency with a translation probability, as shown in Fig. 1c. For an initial insertion of a signal portion, the probability of persisting in the signal frequency, similar to transmittance in a spatial beam splitter, is ${\rm{co}}{{\rm{s}}}^{2}({\rm{gL}})$, while that of translating to the idler frequency, like reflectance in a spatial beam splitter, is ${\rm{si}}{{\rm{n}}}^{2}({\rm{gL}})$. Control of the splitting ratio in the frequency domain is achievable by changing the BS-FWM pump power. Consequently, the BS-FWM effect enables the implementation of frequency beam splitters analogous to spatial beam splitters^21,33^. In this work, we have taken the frequency beam splitter a step further by creating the NOON state in the frequency domain, which is a significant milestone in quantum optics, for the first time to the best of our knowledge.

Our proposed concept of NOON-state interference in the frequency domain is illustrated in Fig. 1d. ① Instead of feeding two identical single photons into the two input ports of a spatial beam splitter, we introduce two single photons—one embodying the signal frequency, the other the idler frequency—into a frequency beam splitter. Here, different frequencies entering the frequency beam splitter correspond to the two input ports of the spatial beam splitter. A translation probability of 50% induces the HOM effect in the frequency domain, and the resultant NOON state of *N* = 2 in the frequency domain signifies the frequency two-photon bunching either in the signal frequency or the idler frequency. ② We control the relative phase of the NOON state by adjusting the position of the secondary frequency beam splitter as the length difference between the signal and idler wavelengths causes a relative phase shift between them. ③ The final state, after traversing the secondary frequency beam splitter, exhibits periodic two-photon bunching and anti-bunching effects in the frequency domain, influenced by the relative phase. The bunching effect can be observed by placing a filter to separate the signal and idler photons. Therefore, we are able to implement the NOON-state interference in the frequency domain via Bragg scattering four-wave mixing.

In the interferometer depicted in Fig. 1d, the output state after the second frequency beam splitter is given by

3 $$\left|{{\rm{\psi }}}_{{\rm{NOON}}}\right\rangle =\frac{1}{\sqrt{2}}{{\rm{e}}}^{{\rm{i}}(\Delta {\rm{\phi }}+{\rm{\phi }})}\sin \left(\Delta {\rm{\phi }}\right){\left|2\right\rangle }_{{\rm{s}}}{\left|0\right\rangle }_{{\rm{i}}}-\frac{1}{\sqrt{2}}{{\rm{e}}}^{{{i}}\left(\Delta {\rm{\phi }}-{\rm{\phi }}\right)}\sin \left(\Delta {\rm{\phi }}\right){\left|0\right\rangle }_{{\rm{s}}}{\left|2\right\rangle }_{{\rm{i}}}-{{\rm{e}}}^{{{i}}\Delta {\rm{\phi }}}\cos \left(\Delta {\rm{\phi }}\right){\left|1\right\rangle }_{{\rm{s}}}{\left|1\right\rangle }_{{\rm{i}}}$$

where ${\left|{{N}}\right\rangle }_{{\rm{m}},{\rm{out}}}$ ($\tt {{m}}={{s}},{{i}}$) represents the Fock states (*N*-photon-number states) at the output signal and idler modes, respectively. The relative phase Δ*ϕ* is defined as $\Delta {{\phi }}=2{{\pi }}\Delta {{f}}\,{{L}}/{{c}}$, where Δ*f* is the frequency difference between the signal and idler modes ($\Delta {{f}}={{{f}}}_{{\rm{s}}}-{{{f}}}_{{\rm{i}}}$). The parameter L represents the distance between the two frequency beam splitters. This formulation reveals the oscillating two-photon bunching probability with a period of ${{c}}/(2\Delta {{f}})$. In contrast, when classical light or single photons are injected into the same interferometer, the oscillation period doubles, becoming ${\rm{c}}/\Delta {{f}}$. Notably, while the coefficient ϕ in Eqs. (1) and (2) is necessary for the beam splitter operators, it does not influence the measurement outcomes for the HOM effect, NOON-state interference, or single-photon interference in the frequency domain. See Supplementary Information, Sec. I, for details about analytic calculations.

**Fig. 2:**
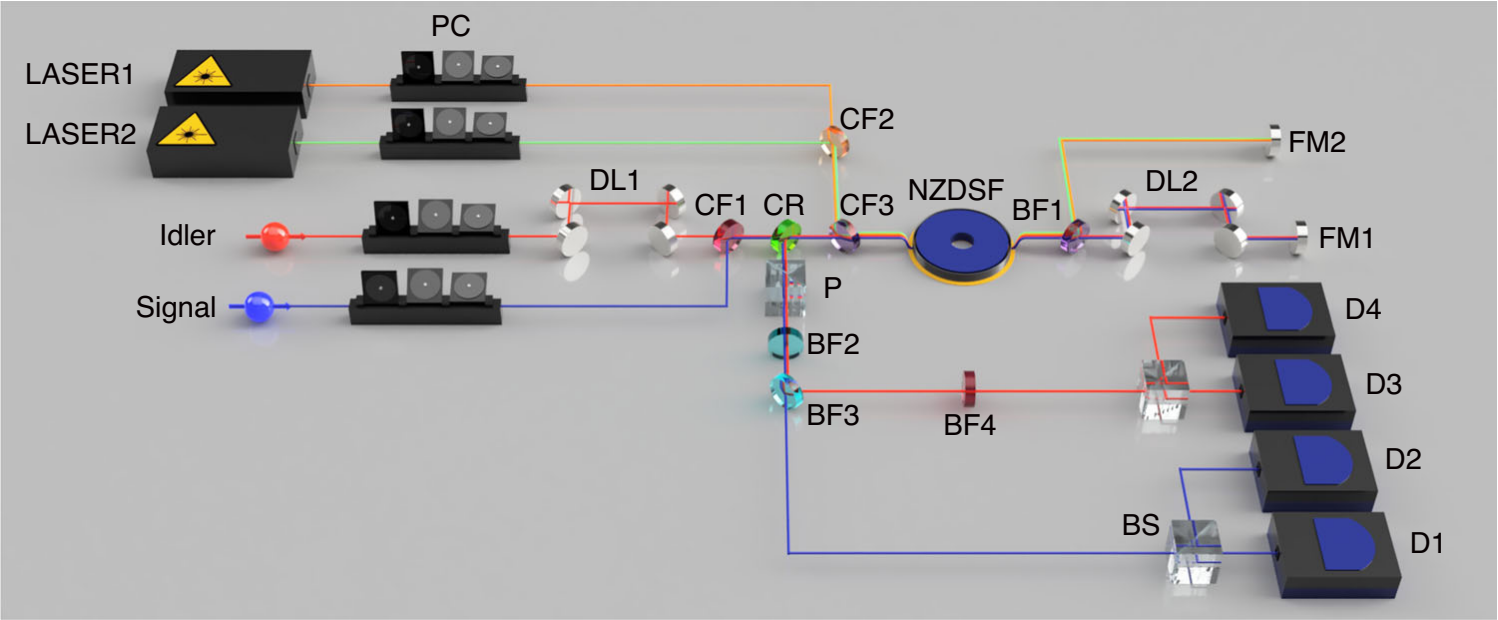
**Experimental setup of the NOON-state interference**. PC polarization controller, DL delay line, CF combining filter, CR circulator, NZDSF non-zero dispersion-shifted fiber, BF bandpass filter, FM Faraday mirror, P polarizer, BS beam splitter, D superconducting nanowire single-photon detector

Our experimental setup for observing NOON-state interference in the frequency domain is shown in Fig. 2. Non-degenerate photon pairs are created via spontaneous four-wave mixing in a 200-m-long single-mode fiber (Corning, SMF-28). The pump laser has a center wavelength of 1269.50 nm and temporal duration of 0.1 ns. Details about the photon-pair generation is described in the Methods section. The signal and idler photons have center wavelengths of 1267.89 nm and 1271.11 nm, respectively, with a bandwidth of about 0.7 nm. After combining them with a combining filter (CF1), they are introduced to the frequency beam splitter. The relative delay between them is controlled using a delay line (DL1).

The two BS-FWM pump lasers (LASER1 and LASER2) have wavelengths of 1551.16 nm (193.27 THz) and 1546.36 nm (193.87 THz), yielding a translation frequency of 600 GHz, and both of them have a repetition rate of 20 MHz and a temporal duration of 0.5 ns. After combining the two BS-FWM pump lasers using a combining filter (CF2) and equalizing their power, they are synchronized with the photon-pair generation pump electronically. The generated photon pairs and BS-FWM pumps are directed through a 100-m-long non-zero dispersion-shifted fiber (NZDSF) with a ZGVD wavelength of 1401 nm, where the BS-FWM effect with 50% conversion efficiency serves as a frequency beam splitter, facilitating the HOM effect in the frequency domain. The temporal overlaps and polarization direction between the pair photons and BS-FWM pumps are optimized. After the first pass through the frequency beam splitter, the two photons possess identical frequencies, either at the signal or idler frequency, creating the *N* = 2 NOON state in the frequency domain. The photon pairs and BS-FWM pumps are separated by a bandpass filter (BF1) and reflected back by Faraday mirrors (FM1 and FM2), enabling their backward propagation through the NZDSF. Additionally, we adjust the optical path length (DL2) to ensure temporal overlap between the pair photons and BS-FWM pumps during the backward propagation.

The relative phase within the NOON state is controlled by a delay line (DL2). Note that both signal and idler photons traverse a single-mode fiber. Given the wavelength discrepancy between the signal (1267.89 nm) and idler (1271.11 nm) photons, which is approximately 3.22 nm, a path length variation equivalent to the idler wavelength would generate a relative phase shift of approximately 0.253% between the signal and idler. A 0.5-mm path length variation to DL2 corresponds to one idler wavelength of the relative phase shift. This path length variation is identical to a temporal change of 1.67 ps, much smaller than the pump duration of BS-FWM, guaranteeing still good temporal overlap between the BS-FWM pumps and the pair photons. Under our experimental conditions, where the pair photons pass the DL2 twice, a delay-line change of about 0.25 mm would cause one period in a classical interference pattern.

A circulator (CR) extracts the backward photons from the NOON-state interferometer. BS-FWM pumps are initially filtered out by a combining filter (CF3), and any residual pumps are further suppressed by a bandpass filter (BF2) with a bandwidth of 16.9 nm centered at 1270 nm, achieving a rejection rate exceeding −120 dB. The signal and idler photons are separated by additional bandpass filters (BF3 and BF4), each with a bandwidth of 0.7 nm centered at 1267.89 nm and 1271.11 nm, respectively. At each path, a beam splitter along with two superconducting nanowire single-photon detectors (SNSPDs; Scontel, HED model) facilitate the post-selection of two-photon events. Given that the SNSPDs are optimized for C-band photons, their measurement efficiency diminishes to around 40% in the O-band. Furthermore, their dark count rates are maintained around 100 Hz. The data is collected via a time-correlated single photon counting (TCSPC, Swabian instruments) module with a coincidence window of 0.3 ns. The total transmission of the quantum frequency translation setup is about 59% (−2.3 dB), including the transmissions from the combining filter (CF3: 93%, −0.3 dB), NZDSF and bandpass filter (NZDSF and BF1: 87%, −0.6 dB), and noise block filter for the BS-FWM effect (BF2: 72%, −1.4 dB). Note that the intrinsic transmission from a 100-m-long NZDSF is negligible (~99%, −0.05 dB).

In our study, we meticulously characterize the splitting ratios of a frequency beam splitter for both the forward and backward directions. The splitting ratio can be determined by monitoring the converted and non-converted signal single-photon counts while we isolate the idler photons. First, we ensure BS-FWM operates in a single direction, achieved by carefully adjusting the optical delay (DL2) and BS-FWM pump arrival time. The DL2 is varied by about 4.5 ns from its optimum position for both the forward and backward BS-FWM. To activate BS-FWM only in the forward direction, the pumps and signal photons arrive at the frequency beam splitter at their first passing. Due to the double passing of the 4.5-ns delay, the photons and BS-FWM pumps are separated by 9.0 ns, which is 18 times longer than the BS-FWM pump duration, causing no BS-FWM in backward propagation. Similarly, to achieve only backward BS-FWM, we postpone the BS-FWM pumps electronically by 9.0 ns. Then, the pumps coincide with signal photons at the frequency beam splitter at the second passing.

**Fig. 3:**
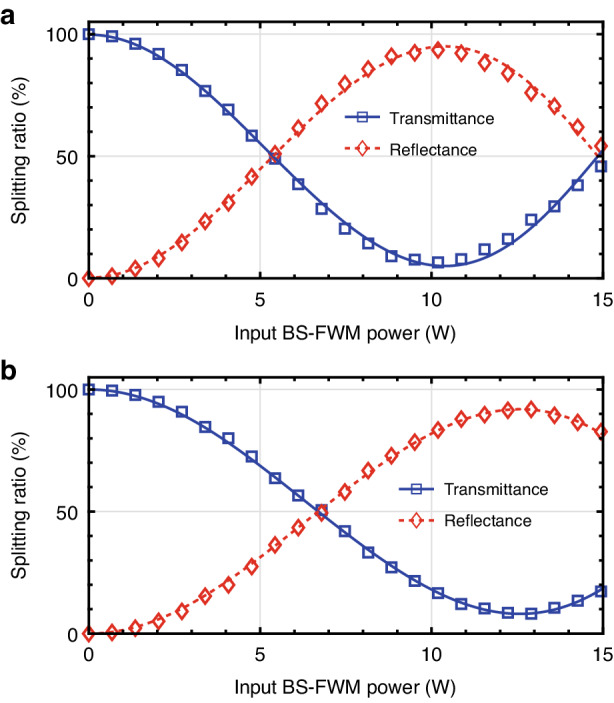
**Splitting ratio in a frequency beam splitter for forward and backward propagation**. The variation in splitting ratios as a function of input BS-FWM pump power is depicted for **a** forward and **b** backward propagation. The experimentally measured transmittance and reflectance are presented by blue squares and red diamonds, respectively. The fitting of these data points is achieved through solid blue lines for transmittance and dashed red lines for reflectance, both of which are modeled as envelopes of sinusoidal functions

The measured splitting ratio in the forward and backward direction is shown in Fig. 3. The transmittance (blue squares, *T*) of the splitting ratio is defined as the ratio between non-converted photon counts and total (converted and non-converted) photon counts at each pump power. As we assume that the frequency translation occurs only between signal and idler wavelengths, the reflectance (red diamonds) of the splitting ratio is ${{R}}=1-{{T}}$. In reality, a small percentage of photons are diverted due to scattering into higher-order modes, but this loss is minor and does not significantly affect the calculation of the splitting ratio^30^.

Here, the background counts are measured at the signal and idler channels by blocking the input photons and then subtracted from the raw data when calculating the splitting ratio. See Supplementary Information, Sec. II, for details on the measurement of the splitting ratio. The solid blue and dashed red lines represent the fitted curves of the envelopes, which exhibit a sinusoidal waveform. As illustrated in Fig. S1, the maximum depletion rates are achieved at pump powers of 10.4 W and 12.7 W for the forward and backward directions, respectively. These discrepant pump powers indicate the pump power loss during the journey through the interferometer, which is approximately 18% loss (−0.85 dB).

We observe the maximum translation efficiencies of 85.9 ± 1.6% and 81.5 ± 1.5% for the forward and backward directions, respectively. See Supplementary Information, Sec. II, for details on the translation efficiencies. These high efficiencies are attributed to the dispersion property of the NZDSF, which constraints the leakage to the unintended frequency bands and increases the translation efficiencies, given by the narrow phase-matching bandwidth of 1.32 nm^31^. We expect that higher translation efficiencies are available by engineering the dispersion property of optical fibers. Additionally, In the quantum frequency translation process, the generation of noise photons via spontaneous four-wave mixing^34^ and spontaneous Raman scattering is negligible. This is attributed to the separation of more than 270 nm between the BS-FWM pumps and signal/idler photons. See Supplementary Information, Sec. II, for the measured noise counts.

**Fig. 4:**
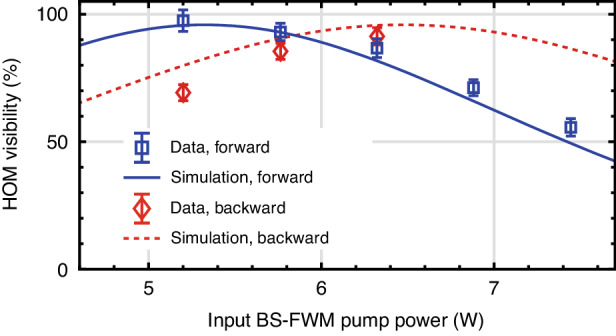
**HOM interference using the frequency beam splitter**. Blue squares and red diamonds represent the net visibilities for the forward and backward directions of the HOM effect, respectively. The error bars are calculated from the fit coefficients and confidence bounds of the HOM interference. Solid blue and red dashed curves are the simulation results

Figure 4 represents the net visibility of the HOM interference versus the input BS-FWM pump power. Blue squares and red diamonds depict the net visibilities for the forward and backward directions of BS-FWM, respectively. The solid blue and dashed red lines correlate to the numerical simulations using a Green-function method^30,35^, modeling the effect of BS-FWM^36,37^ as an input-output relation. See Supplementary Information, Sec. III.A. for the simulation method and Sec. IV for the frequency-domain HOM interference without accidental subtraction. The experimental results match well with the simulation results.

Creating a NOON interferometer requires two 50:50 beam splitters. Due to a NOON-interferometer transmission of 82% (-0.85 dB) for the BS-FWM pumps, the HOM visibility for forwarding and backward propagation peaks at different powers of 5.20 W and 6.32 W, respectively, where the splitting ratio is close to 50%, and their maximum net (raw) visibility is 97.5 ± 4.2% (90.5 ± 3.8%) and 91.3 ± 3.4% (83.9 ± 3.0%), respectively, as seen in Fig. 4. As the 82% transmission incurred through the NOON interferometer makes it difficult to obtain a 50% transmittance for both the forward and backward directions, we utilize an input BS-FWM power of 5.76 W, achieving a transmittance of 45% in the forward direction, leading to a 60% transmittance in the backward direction, where the corresponding net (raw) visibilities are 93.0 ± 3.4% (86.5 ± 3.2%) and 85.5 ± 3.2% (80.2 ± 2.9%), respectively. These unbalanced splitting ratios will lead to non-ideal visibility in the NOON-state interference.

**Fig. 5:**
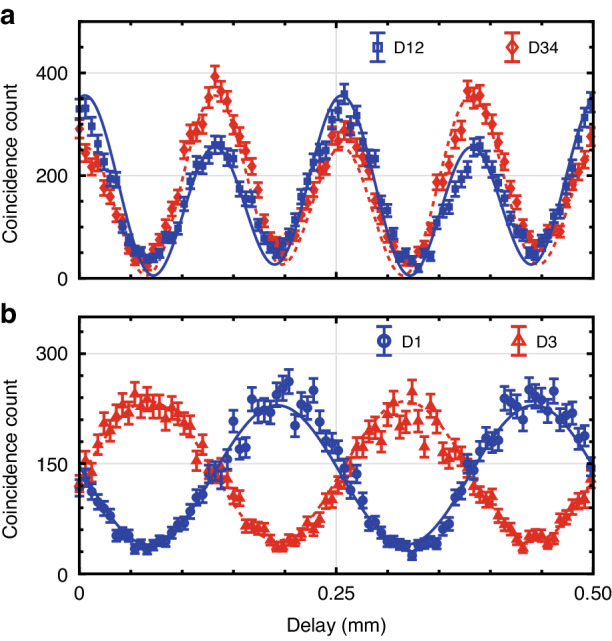
Frequency-domain NOON-state interference. **a** The true coincidence counts versus the temporal delay (DL2) for the NOON-state interference (*N* = 2). The blue and red points indicate the true coincidence counts of the SNSPDs D1 & D2 and D3 & D4, respectively, which enables post-select two-photon events. The solid blue and dashed red lines are simulation results. **b** The true coincidence counts versus the temporal delay (DL2) for the single-photon interference. The blue and red points represent the true coincidence counts of the SNSPDs D1 & D5 and D3 & D5. The solid blue and dashed red lines are simulation results. The error bars are calculated assuming Poissonian statistics of the detection

The frequency-domain NOON-state interference and single-photon interference patterns are shown in Fig. 5. The NOON interference is gauged using the experimental setup described in Fig. 2. Figure 5a depicts the NOON-state interference, where the blue squares and red diamonds indicate the true coincidence counts between the SNSPDs D1 & D2 and D3 & D4, respectively. Each point is accumulated for 60 s. The solid blue and dashed red lines are the simulation results, which align closely with the experimental findings. See Supplementary Information, Sec. III.B, for the simulation method of the frequency-domain NOON-state interference. Notably, the HOM interference in Fig. 4 and the two-fold enhanced quantum resolution in Fig. 5 is the signature of the generation of the 2002 state, $\left|{{\psi }}\right\rangle =\frac{1}{\sqrt{2}}\left({\left|2\right\rangle }_{{\rm{s}}}{\left|0\right\rangle }_{{\rm{i}}}+{\left|0\right\rangle }_{{\rm{s}}}{\left|2\right\rangle }_{{\rm{i}}}\right)$, commonly referred to as the photon number path entangled state. Our results demonstrate the achievement of super-resolution within this framework. However, it is important to note that super-sensitivity was not observed, which is attributable to system losses. See Supplementary Information, Sec. V for detailed calculations about Fisher Information and super-sensitivity.

In comparison, Fig. 5b presents the single-photon interference, which has been implemented using a slightly modified version of the setup depicted in Fig. 2. Rather than deploying both signal and idler photons into the interferometer, the idler photon is employed as a trigger photon using a heralding detector D5, which is not shown in Fig. 2, and the heralded signal photon is directed toward the interferometer. Two beam splitters preceding the detectors D1 and D3 are removed to enhance the count rates. The blue circles and red triangles indicate the true coincidence counts between the SNSPD D1 & D5 and D3 & D5, respectively. Each data point is acquired for one second. The solid blue and dashed red lines are the theoretical results, which correlate closely with the experimental data. See Supplementary Information, Sec. III.C for the simulation method of the single-photon interference and Sec. VI for the measurement results without subtracting the accidental counts. By fitting the oscillations with a sinusoidal function, we extracted the oscillation periods as 0.125 ± 0.001 mm for the NOON state and 0.248 ± 0.001 mm for the single-photon state. Theoretically, these periods are calculated to be 0.125 mm and 0.250 mm, respectively, derived using the relation ${\rm{Period}}={{c}}/(2{{N}}\Delta {{f}})$. The factor of two in this formula arises from the double pass of the delay line in our experiment. The experimentally determined oscillation periods align well with these theoretical expectations. While our current measurements focus on the 2002-interference pattern, our experiment setup is adaptable for the measurement of the 4004-interference pattern via post-selection^9,11^.

**Fig. 6:**
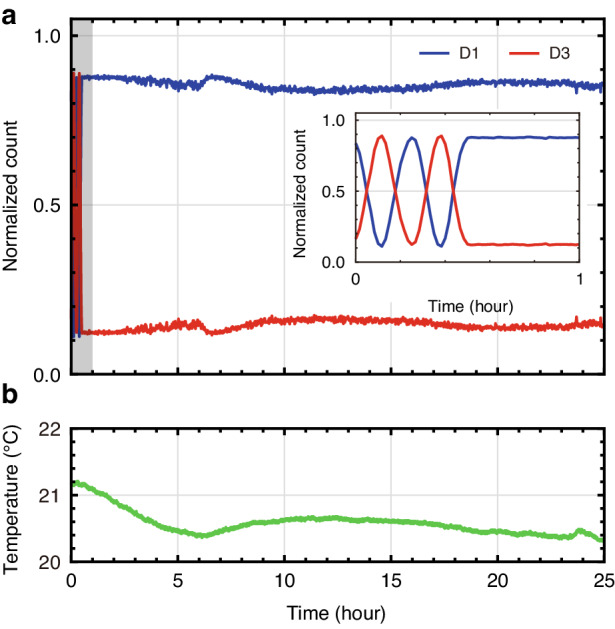
**Stability analysis of the frequency-domain NOON-state interferometer. a** Normalized coincidence counts over 25 h-time periods using heralded signal-photons. The blue and red curves represent the coincidence detections between the SNSPDs D1 & D5 and D3 & D5, respectively. The inset provides a magnified view over a 1-hour period, which is shown as a dashed gray box. **b** Temperature measurements from our laboratory, captured during the identical time window of the stability test

Furthermore, our interferometer shows outstanding stability due to the use of a single-mode spatial path. Two frequency modes propagate a single-mode fiber, and this guarantees that the phase shifts of the two frequency modes will be almost identical, effectively neutralizing the phase difference between them. Such attributes enable the frequency-domain interferometer with exceptional stability. Figure 6a illustrates the experimental results of a single-photon interferometer stability test conducted over a 25-h period. The blue and red curves represent the normalized coincidence counts between SNSPDs D1 & D5 and D3 & D5, respectively, where D5 is the heralding detector. Each data point is accumulated in one-minute intervals. For the first 0.5 h, we introduce a two-cycle of phase change by tuning the temporal delay line (DL2). Then, for the remainder of the test duration, this path-length variation is halted. The inset of Fig. 6a represents an expanded view of Fig. 6a, captured during the first hour of measurement. The measured interference patterns display remarkable stability. Of particular note, this experiment was performed on an unfloated optical table with a hundred meters of optical fibers. Moreover, the setup was located under an air conditioner without any active temperature/phase control or wind protection. The expected temperature stability of our interferometer, including the double-pass propagation of the 100-m NZDSF and additional meters of single-mode fibers, is roughly $\sim 0.4\,{\rm{\pi }}/^\circ {\rm{C}}$, which is calculated as 1.4 × 10^−^^3^ ps/nm/km/°C × 3.2 nm × 0.2 km × 2πc/1270 nm. This calculation is based on a chromatic dispersion thermal coefficient of 1.4 × 10^−3^ ps/nm/km/°C, derived from measurements on a single-mode fiber at 1550 nm^38^. Figure 6b represents the measured temperature stability within our laboratory during the same time frame as our stability test. The average temperature is 20.6 ± 0.2 °C throughout this 25-h period. Under these conditions, the frequency-domain interferometer exhibits unprecedented stability.

In conclusion, we demonstrated the pioneering NOON-state interference in the frequency domain by employing frequency beam splitters and non-degenerate photon pairs. The NOON state’s relative phase is controlled by a delay line, resulting in two-photon interference. The resulting observations include the two-photon frequency bunching and anti-bunching effects depending on the relative phase between the two frequency modes, two-fold enhancement in the oscillation period compared to that of a single-photon interference, and unprecedented stability of the interferometer.

## Discussion

The NOON state exhibits a non-sinusoidal interference pattern, while the single-photon state shows a sinusoidal pattern. In addition, both patterns exhibit non-ideal visibility. The following three factors contribute to this non-sinusoidal pattern and non-ideal visibility: (1) asymmetricity in the joint-spectral intensity, (2) dispersion effect of the NZDSF causing a walk-off between the two photons, and (3) unbalanced BS-FWM pump powers for forward and backward propagation. These three effects can be mitigated by the following strategies: (1) Reducing the length of the nonlinear medium (SMF) for the photon-pair generation to relax the phase-matching condition^34^. (2) Placing a dispersion compensation component to avoid the walk-off effect, such as a pair of gratings or an optical Bragg filter. (3) Amplifying the BS-FWM pumps to compensate for the NOON interferometer insertion loss. See Supplementary Information, Sec. III, for details on these three factors contributing to non-ideal visibility.

In this study, a high pump power of up to 15 W was utilized to compensate for the low nonlinearity coefficient of the optical fiber. Despite this, the generation of noise photons remained negligibly low, posing no significant issue. In addition, this high pump power requirement significantly decreases when utilizing on-chip platforms. A demonstration in a silicon waveguide reveals an efficiency of 12% with the BS-FWM pump power of 1.6 mW^39^. Additionally, silicon nitride resonators report an efficiency of 60% with a pump power of 60 mW^40^. This subject is fascinating but not within our current research scope. It is, however, a prime candidate for our future research projects.

With its scalability to high-dimensional multi-frequency states and the potential for miniaturization through single-mode propagation, quantum information processing in the frequency domain has attracted significant attention, especially in the context of quantum communications^41^. As shown in this study, frequency-domain quantum information processing highlights the compatibility with optical fiber networks, in addition to demonstrating the remarkable stability of our interferometer, a crucial resource in both fundamental research and application^42–44^, including quantum communications^45^ and linear quantum computing^46^.

Owing to the excellent stability and scalability of our quantum states, our work can be extended to implement high-dimensional quantum key distribution (QKD) through deployed fiber networks. This method enhances information efficiency and may extend the maximum transmission distance compared to conventional two-dimensional QKD systems^47^. Notably, high-dimensional states in the frequency domain can be readily generated by inserting optical filters or a Fabry–Perot cavity after the creation of photon pairs^41^. Quantum simulators can be investigated within the frequency domain, including quantum random walks^48^ and Boson sampling^46^. By varying the number of BS-FWM pumps and adjusting the relative phase and power between them, we can explore a variety of quantum-circuit configurations and interesting phenomena, including non-local hopping^28,29^. These features may offer insights into areas not readily accessible through conventional path-mode schematics. Finally, our NOON-state interferometer, exploiting the quantum interference across different frequencies, is capable of measuring wavelength-dependent phase shifts. This feature holds the potential to implement quantum quantitative phase spectroscopy, which investigates the properties of organisms or cells, especially those with extremely low damage thresholds^21,49^.

## Methods

### Preparation of the photon pair

A non-degenerate photon pair is created via spontaneous four-wave mixing within a 200-m-long single-mode fiber (Corning, SMF-28), mediated by a pump laser under the relaxed phase-matching condition^34^. The pump laser features a central wavelength of 1269.50 nm, a duration of 100 ps, and a peak power of about 1 W. The laser operates at a repetition rate of 20 MHz, electronically synchronized with the BS-FWM pump lasers. We employ two strategies to reduce the noise photons resulting from spontaneous Raman scattering. First, we cool the single-mode fiber by submerging it in liquid nitrogen, thereby reducing the population of excited state phonons. Second, we attach a polarizer to the output of the single-mode fiber and align the polarization direction of the pump to that of the polarizer. This is because the polarization state of the photon pair is parallel to that of the pump laser, while noise photons are unpolarized. The pump, signal, and idler photons are separated by bandpass filters with a bandwidth of 0.7 nm and centered at 1269.50, 1267.89, and 1271.11 nm, respectively. The setup ensures a pump rejection ratio exceeding 120 dB. The resultant coincidence-to-accidental ratio for the photon pair is 22.2 ± 1.1.

### Supplementary information

Supplemental Material: NOON-state interference in the frequuency domain
