# Supplementary material for: NOON-state interference in the frequency domain
Source: Light Sci Appl. 2024 Apr 15;13:90. doi: 10.1038/s41377-024-01439-9 (PMC11018870; doi:10.1038/s41377-024-01439-9)
Supplement: Supplementary file 1 — Supplemental Material: NOON-state interference in the frequuency domain [file 41377_2024_1439_MOESM1_ESM.pdf]

Supplementary Information for NOON-state interference in the frequency domain

Dongjin Lee, Woncheol Shin, Sebae Park, Junyeop Kim, and Heedeuk Shin*
Department of Physics, Pohang University of Science and Technology (POSTECH), Pohang, 37673, South Korea
(Dated: March 16, 2024)

I. BACKGROUND THEORY

In this section, we will derive analytic solutions of the Hong-Ou-Mandel (HOM) effect, NOON-state interference, and single-photon interference in the frequency domain. We consider the single-mode inputs to derive the analytic solutions. The solutions for these effects with pulsed mode inputs will be calculated in Sec. III.

A. Frequency-domain HOM interference

As we discussed in the main text, the evolution of the annihilation mode operators via Bragg scattering four-wave mixing (BS-FWM) is described by the following equation[1–3],

$$\begin{bmatrix}\hat{a}_{\mathrm{s,out}}\\ \hat{a}_{\mathrm{i,out}}\end{bmatrix} = \begin{bmatrix}\cos(gL) & ie^{i\phi}\sin(gL)\\ ie^{-i\phi}\sin(gL) & \cos(gL)\end{bmatrix}\begin{bmatrix}\hat{a}_{\mathrm{s,in}}\\ \hat{a}_{\mathrm{i,in}}\end{bmatrix}. \tag{S1}$$

Here, the coefficient g is defined by $g \equiv \gamma P$, where γ and P denote the nonlinear coefficient of the nonlinear medium and the total pump power of BS-FWM, respectively. The term ϕ denotes the phase difference between the two pump beams. This equation resembles the transformations in beam splitters, allowing us to interpret BS-FWM as a frequency beam splitter[3,4]. While the coefficient ϕ appears unusual, we will confirm that this coefficient does not affect the measured outcomes. For ease of calculation, we rewrite Eq. (S1) by representing the input-creation operators in terms of the output-creation operators,

$$\begin{bmatrix}\hat{a}^{\dagger}_{\mathrm{s,in}}\\ \hat{a}^{\dagger}_{\mathrm{i,in}}\end{bmatrix} = \begin{bmatrix}\cos(gL) & ie^{-i\phi}\sin(gL)\\ ie^{i\phi}\sin(gL) & \cos(gL)\end{bmatrix}\begin{bmatrix}\hat{a}^{\dagger}_{\mathrm{s,out}}\\ \hat{a}^{\dagger}_{\mathrm{i,out}}\end{bmatrix}. \tag{S2}$$

When inserting two single photons of different frequencies (signal and idler) into a frequency beam splitter, the state is transformed as

$$\begin{aligned}\hat{a}^{\dagger}_{\mathrm{s,in}}\hat{a}^{\dagger}_{\mathrm{i,in}}|0\rangle &= [\cos(gL)\hat{a}^{\dagger}_{\mathrm{s,out}} + ie^{-i\phi}\sin(gL)\hat{a}^{\dagger}_{\mathrm{i,out}}][ie^{i\phi}\sin(gL)\hat{a}^{\dagger}_{\mathrm{s,out}} + \cos(gL)\hat{a}^{\dagger}_{\mathrm{i,out}}]|0\rangle\\ &= ie^{i\phi}\cos(gL)\sin(gL)\hat{a}^{\dagger}_{\mathrm{s,out}}\hat{a}^{\dagger}_{\mathrm{s,out}}|0\rangle + ie^{-i\phi}\cos(gL)\sin(gL)\hat{a}^{\dagger}_{\mathrm{i,out}}\hat{a}^{\dagger}_{\mathrm{i,out}}|0\rangle + \left[\cos^2(gL) - \sin^2(gL)\right]\hat{a}^{\dagger}_{\mathrm{s,out}}\hat{a}^{\dagger}_{\mathrm{i,out}}|0\rangle.\end{aligned} \tag{S3}$$

At the splitting ratio of 50% ($gL = \pi/4$), the HOM effect in the frequency domain can be observed, as indicated by

$$\begin{aligned}\hat{a}^{\dagger}_{\mathrm{s,in}}\hat{a}^{\dagger}_{\mathrm{i,in}}|0\rangle &= \frac{i}{2}e^{i\phi}\hat{a}^{\dagger}_{\mathrm{s,out}}\hat{a}^{\dagger}_{\mathrm{s,out}}|0\rangle + \frac{i}{2}e^{-i\phi}\hat{a}^{\dagger}_{\mathrm{i,out}}\hat{a}^{\dagger}_{\mathrm{i,out}}|0\rangle\\ &= \frac{i}{\sqrt{2}}e^{i\phi}|2\rangle_{\mathrm{s,out}}|0\rangle_{\mathrm{i,out}} + \frac{i}{\sqrt{2}}e^{-i\phi}|0\rangle_{\mathrm{s,out}}|2\rangle_{\mathrm{i,out}},\end{aligned} \tag{S4}$$

where $|N\rangle_{\mathrm{m,out}}$ ($\mathrm{m} = \mathrm{s,i}$) represent the Fock states (N-photon-number states) at the output signal and idler modes, respectively.

* heedeukshin@postech.ac.kr

B. Frequency-domain NOON-state interference

The function of the interferometer in Fig. 1**d** can be represented by the following matrix multiplications,

$$\begin{bmatrix} \hat{a}^\dagger_{\mathrm{s,in}} \\ \hat{a}^\dagger_{\mathrm{i,in}} \end{bmatrix} = \frac{1}{2} \begin{bmatrix} 1 & ie^{-i\phi} \\ ie^{i\phi} & 1 \end{bmatrix} \begin{bmatrix} \exp[-\frac{i2\pi f_{\mathrm{s}} L}{c}] & 0 \\ 0 & \exp[-\frac{i2\pi f_{\mathrm{i}} L}{c}] \end{bmatrix} \begin{bmatrix} 1 & ie^{-i\phi} \\ ie^{i\phi} & 1 \end{bmatrix} \begin{bmatrix} \hat{a}^\dagger_{\mathrm{s,out}} \\ \hat{a}^\dagger_{\mathrm{i,out}} \end{bmatrix}, \tag{S5}$$

where the first and third terms represent the frequency beam splitters, and the second term indicates the phase term, introduced by the distance between the two splitters. We assumed that the splitting ratios of the beam splitters are 50% ($gL = \pi/4$). The terms f_{s} and f_{i} are the frequencies of the signal and idler modes, respectively, and L is the distance between the two splitters. The resultant result from Eq. (S5) is expressed as

$$\begin{aligned} \begin{bmatrix} \hat{a}^\dagger_{\mathrm{s,in}} \\ \hat{a}^\dagger_{\mathrm{i,in}} \end{bmatrix} &= \frac{1}{2} \exp\left[-\frac{i2\pi f_{\mathrm{s}} L}{c}\right] \begin{bmatrix} 1 & ie^{-i\phi} \\ ie^{i\phi} & 1 \end{bmatrix} \begin{bmatrix} 1 & 0 \\ 0 & e^{i\Delta\phi} \end{bmatrix} \begin{bmatrix} 1 & ie^{-i\phi} \\ ie^{i\phi} & 1 \end{bmatrix} \begin{bmatrix} \hat{a}^\dagger_{\mathrm{s,out}} \\ \hat{a}^\dagger_{\mathrm{i,out}} \end{bmatrix} \\ &= \frac{1}{2} \exp\left[-\frac{i2\pi f_{\mathrm{s}} L}{c}\right] \begin{bmatrix} 1 - e^{i\Delta\phi} & ie^{-i\phi}(1 + e^{i\Delta\phi}) \\ ie^{i\phi}(1 + e^{i\Delta\phi}) & -1 + e^{i\Delta\phi} \end{bmatrix} \begin{bmatrix} \hat{a}^\dagger_{\mathrm{s,out}} \\ \hat{a}^\dagger_{\mathrm{i,out}} \end{bmatrix}, \end{aligned} \tag{S6}$$

where $\Delta\phi = 2\pi\Delta f L/c$ and $\Delta f = f_{\mathrm{s}} - f_{\mathrm{i}}$. We will ignore the global phase factor for further calculations, as it does not influence the measurement results. Inserting two single photons (signal and idler photons) into the interferometer, the output state is given by

$$\begin{aligned} \hat{a}^\dagger_{\mathrm{s,in}} \hat{a}^\dagger_{\mathrm{i,in}} |0\rangle &= \frac{i}{4} e^{i\phi} [1 - e^{2i\Delta\phi}] \hat{a}^\dagger_{\mathrm{s,out}} \hat{a}^\dagger_{\mathrm{s,out}} |0\rangle - \frac{i}{4} e^{-i\phi} [1 - e^{2i\Delta\phi}] \hat{a}^\dagger_{\mathrm{i,out}} \hat{a}^\dagger_{\mathrm{i,out}} |0\rangle - \frac{1}{2} [1 + e^{2i\Delta\phi}] \hat{a}^\dagger_{\mathrm{s,out}} \hat{a}^\dagger_{\mathrm{i,out}} |0\rangle \\ &= \frac{1}{\sqrt{2}} e^{i(\Delta\phi + \phi)} \sin(\Delta\phi) |2\rangle_{\mathrm{s,out}} |0\rangle_{\mathrm{i,out}} - \frac{1}{\sqrt{2}} e^{i(\Delta\phi - \phi)} \sin(\Delta\phi) |0\rangle_{\mathrm{s,out}} |2\rangle_{\mathrm{i,out}} - e^{i\Delta\phi} \cos(\Delta\phi) |1\rangle_{\mathrm{s,out}} |1\rangle_{\mathrm{i,out}} . \end{aligned} \tag{S7}$$

The oscillation period for the two-photon detection for the NOON-state interference (N = 2) is $c/(2\Delta f)$.

C. Frequency-domain single-photon interference

The single-photon interference can be calculated with Eq. (S6). When a signal photon is injected into the interferometer, the output state is expressed as

$$\begin{aligned} \hat{a}^\dagger_{\mathrm{s,in}} |0\rangle &= \frac{1}{2} [1 - e^{i\Delta\phi}] \hat{a}^\dagger_{\mathrm{s,out}} |0\rangle + \frac{i}{2} e^{-i\phi} [1 + e^{i\Delta\phi}] \hat{a}^\dagger_{\mathrm{i,out}} |0\rangle \\ &= -ie^{i\Delta\phi/2} \sin(\Delta\phi/2) |1\rangle_{\mathrm{s,out}} |0\rangle_{\mathrm{i,out}} + ie^{i\Delta\phi/2 - i\phi} \cos(\Delta\phi/2) |0\rangle_{\mathrm{s,out}} |1\rangle_{\mathrm{i,out}} , \end{aligned} \tag{S8}$$

where the oscillation period is $c/(\Delta f)$, twice as long as that for the NOON-state interference (N = 2).

In conclusion, we have calculated the Hong-Ou-Mandel effect, NOON-state interference, and single-photon interference in the frequency domain. Despite the presence of the coefficient ϕ in Eq. (S1), which differs from typical beam splitter operators, it does not affect the outcome of the measurements.

II. DETAILED INFORMATION ABOUT THE SINGLE-PHOTON TRANSLATION

The single-photon translation of the BS-FWM effect for the forward direction (a) and the backward direction (b) is described in Fig. S1. The experimental setup in Fig. 2 is slightly modified to measure the translation. The signal photons are used as the input photons, while the idler photons are blocked. The delay line (DL2) is increased as much as 4.5 ns to activate the BS-FWM effect only for the single direction. The output of each channel is measured by an SNSPD without the beam splitter to increase the count rate. The blue squares and red diamonds are the measured counts at the signal (input) and idler (translation) wavelength, respectively. To measure the noise of the BS-FWM effect, the background counts are measured at the signal and idler wavelength while the input photons are blocked. As the background counts at each wavelength are similar, we represent the background counts (green triangles) at only the idler (translation) wavelength. Notably, the background counts can be reduced by cooling the DSF with liquid nitrogen, decreasing noise photons originating from spontaneous Raman scattering[3,5]. The solid blue, dashed red, and dotted green lines represent the best-fit curves of the input, translation, and background counts where the fit functions are given by $a\cos^2(bp)+cp$, $a\sin^2(bp)+cp$, and ap, respectively. a, b, c are the fit parameters and p is the total power of the BS-FWM.

We calculate the calibrated counts by compensating the background counts and the relative efficiency between the channels. The relative efficiency of the idler channel is 85% (-0.7 dB) compared to that of the signal channel. With the calibrated counts, we achieve the efficiencies and the fit depletion rates of the BS-FWM effect. The efficiencies are 85.9±1.6% and 81.5±1.5%, and the fit depletion rates are 95.2% and 92.6% for the forward and the backward directions, respectively. The maximum depletion occurs at the different input powers for the forward (10.4 W) and backward directions (12.7 W), respectively, as the NOON-state interferometer introduces the loss to the BS-FWM pump. We attribute the discrepancy between the translation efficiencies to the imperfection in polarization compensation. We use two Faraday mirrors, denoted as FM1 and FM2 in Fig. 2, with the optimized wavelengths of 1310 nm and 1550 nm. While the BS-FWM pumps are within FM1's operation wavelength range at near 1550 nm, but the signal photon is far from FM2's optimized wavelength of 1310 nm. This leads to imperfect polarization compensation, which results in slightly lower translation efficiency for the backward pumping. We expect that the translation efficiency and splitting ratio are identical for the input idler photons. Theoretically, we can identify from

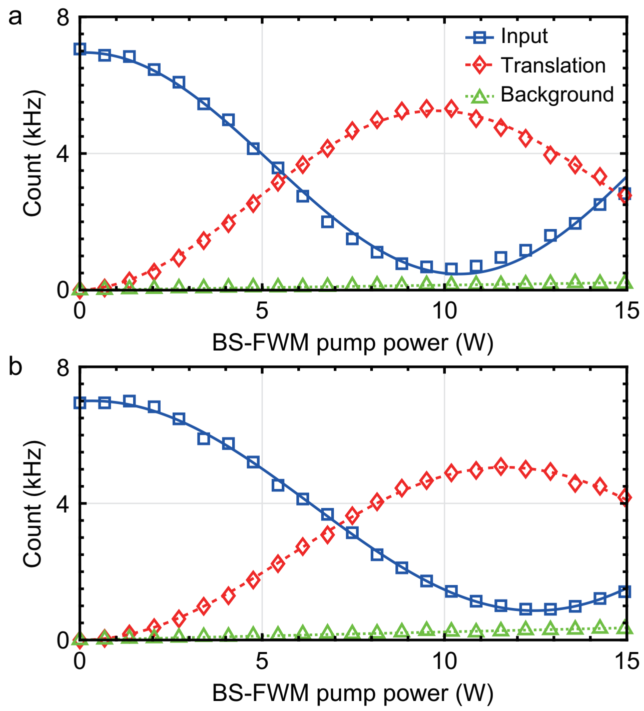

Figure S1. **Single-photon translation of the BS-FWM effect.** The single-photon translation is measured by blocking the idler photons and increasing the delay line (DL2) to allow the quantum frequency translation for the single direction. The blue squares and red diamonds indicate the measured counts at the signal (input) and idler (translation) wavelength, respectively. The background counts (green triangle) represent the measured counts at the idler wavelength while the signal photons are blocked. The lines represent the best-fit curves. **a**, the forward direction. **b**, the backward direction.

Eq. (1) and (2) in the main text that the operator forms for the signal and idler modes are equivalent. Experimentally, Clark et al.[6] demonstrate equivalent translation efficiencies of 99.1%±4.9% and 98.0±5.0% for the input idler and signal photons, respectively.

III. SIMULATION METHOD

A. Frequency-domain HOM interference

We first calculate the joint spectral intensity of the photon-pair generation under our experimental conditions as described in Fig. S2**a**. One of the reasons for having non-unity visibility in Fig. 4, even in theory, is its asymmetrical joint-spectral intensity shape of the photon-pair source under our experimental conditions. See the last of the sections for more details. Note that the asymmetricity in the joint spectral intensity can be resolved by reducing the length of the single-mode fiber (SMF) to relax the phase matching condition[7]. As seen from Fig. S2**b**, we barely observe the asymmetricity by reducing the length of the SMF from 200 m to 50 m.

For convenience in the simulation, Schmidt decomposition is applied to the joint spectral intensity, which represents the correlated function in terms of a linear combination,

$$|\psi_{\rm in}\rangle = \int d\omega_{\rm s} d\omega_{\rm i} F(\omega_{\rm s},\omega_{\rm i}) \hat{a}_{\rm s}^\dagger(\omega_{\rm s}) \hat{a}_{\rm i}^\dagger(\omega_{\rm i}) |0\rangle = \sum_{\rm k} r_{\rm k} \int d\omega_{\rm s} F_{\rm k}(\omega_{\rm s}) \hat{a}_{\rm s}^\dagger(\omega_{\rm s}) \int d\omega_{\rm i} G_{\rm k}(\omega_{\rm i}) \hat{a}_{\rm i}^\dagger(\omega_{\rm i}) |0\rangle \,, \tag{S9}$$

where $F(\omega_{\rm s},\omega_{\rm i})$ represents the joint spectral amplitude and $F_{\rm k}(\omega)$ and $G_{\rm k}(\omega)$ are sets of orthonormal basis, known as Schmidt modes. Schmidt amplitude $r_{\rm k}$ (real number) weights the intensity of each set where $\sum_{\rm k} r_{\rm k}^2 = 1$. $\hat{a}_{\rm s}^\dagger(\omega)$ is a creation operator at the signal mode with a frequency of $\omega + \omega_{\rm s0}$, and $\hat{a}_{\rm i}^\dagger(\omega)$ is a creation operator at the idler mode with a frequency of $\omega + \omega_{\rm i0}$. $\omega_{\rm s0}$ and $\omega_{\rm i0}$ are the central frequencies of bandpass filters (FWHM: 0.7 nm) which are used to refine the joint spectral intensity, where $\omega_{\rm s0} = 2\pi \times 236.45$ THz (1267.89 nm) and $\omega_{\rm i0} = 2\pi \times 235.85$ THz (1271.11 nm), respectively.

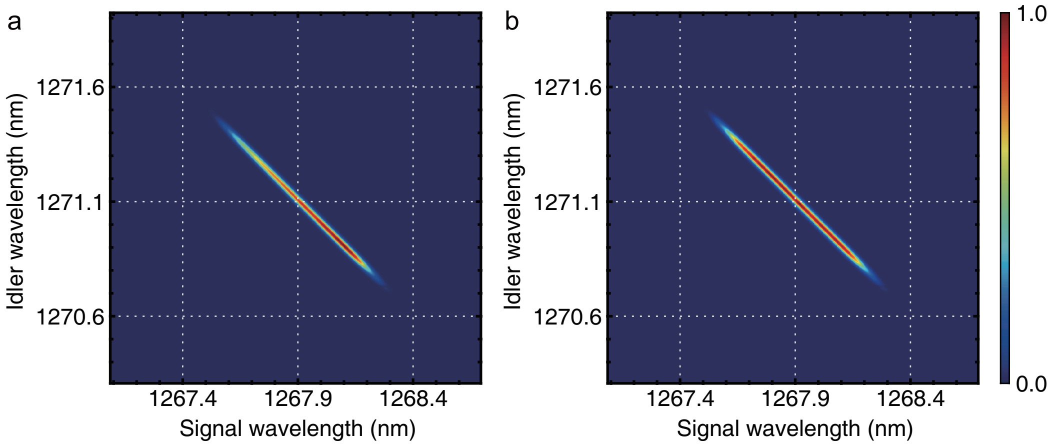

Figure S2. **Simulated joint spectral intensity of the photon pair.** **a**, The simulated conditions are identical to our experimental conditions, where the length of the optical fiber is 200 m. **b**, The length of the optical fiber is shortened to 50 m, but all other conditions remain consistent.

The Bragg scattering four-wave mixing (BS-FWM) effect is numerically implemented with a Green-function method[8], which represents the effect in terms of an input-to-output relation. To construct the Green function, we use Hermite-Gaussian (HG) functions as an orthogonal set of the input amplitudes and calculate the corresponding output amplitudes with coupled equations governing the BS-FWM effect. The coupled equations are derived from the nonlinear Schrödinger equation (NLSE)[8–10]. More details about the construction of the Green function are described in our previous paper[11]. As the length of the SMF is different (150 m) in the previous paper, we use an optimal characteristic length of the HG functions as 29 ps instead of 28 ps to calculate the Green function. The constructed Green function describes the transformation of the creation operators at the signal and idler modes via BS-FWM as follows,

$$\begin{aligned} \hat{a}_{\rm s}^\dagger(\omega) &\to \int d\omega' G_{\rm ss}(\omega,\omega') \hat{a}_{\rm s}^\dagger(\omega') + G_{\rm si}(\omega,\omega') \hat{a}_{\rm i}^\dagger(\omega'), \\ \hat{a}_{\rm i}^\dagger(\omega) &\to \int d\omega' G_{\rm is}(\omega,\omega') \hat{a}_{\rm s}^\dagger(\omega') + G_{\rm ii}(\omega,\omega') \hat{a}_{\rm i}^\dagger(\omega'), \end{aligned} \tag{S10}$$

where $G_{\rm ij}(\omega,\omega')$ $({\rm i,j}={\rm s,i})$ is the Green function which indicates the evolution amplitude describing the frequency component (ω) at the input mode i is translated to the other component (ω') at the output mode j.

Now, we are ready to calculate the frequency-domain HOM interference. The delay line (DL1) introduces a temporal delay (Δt_1) to the idler photon, transforming equation (S9) as below

$$|\psi_{\rm in}\rangle \rightarrow |\psi_{\rm in,1}\rangle = \sum_{\rm k} r_{\rm k} \int d\omega_{\rm s} F_{\rm k}(\omega_{\rm s}) \hat{a}_{\rm s}^{\dagger}(\omega_{\rm s}) \int d\omega_{\rm i} G_{\Delta {\rm t}_1,{\rm k}}(\omega_{\rm i}) \hat{a}_{\rm i}^{\dagger}(\omega_{\rm i})\,|0\rangle\,, \tag{S11}$$

where $G_{\Delta {\rm t}_1,{\rm k}}(\omega) = G_{\rm k}(\omega)\exp[i(\omega_{\rm i0}+\omega)\Delta t_1]$. The BS-FWM process, implementing the role of the frequency beam splitter, evolves the state as

$$|\psi_{\rm out,1}\rangle = \sum_{\rm k} r_{\rm k} \left(\int d\omega_{\rm s} F_{\rm ss,k}(\omega_{\rm s}) \hat{a}_{\rm s}^{\dagger}(\omega_{\rm s}) + F_{\rm si,k}(\omega_{\rm s}) \hat{a}_{\rm i}^{\dagger}(\omega_{\rm s})\right) \times \left(\int d\omega_{\rm i} G_{{\rm is},\Delta {\rm t}_1,{\rm k}}(\omega_{\rm i}) \hat{a}_{\rm s}^{\dagger}(\omega_{\rm i}) + G_{{\rm ii},\Delta {\rm t}_1,{\rm k}}(\omega_{\rm i}) \hat{a}_{\rm i}^{\dagger}(\omega_{\rm i})\right) |0\rangle\,, \tag{S12}$$

where the amplitudes $F_{\rm xy,k}(\omega)$ and $G_{{\rm xy},\Delta {\rm t}_1,{\rm k}}(\omega)$ $({\rm x,y}={\rm s,i})$ indicate $F_{\rm xy,k}(\omega) = \int d\omega' F_{\rm k}(\omega') G_{\rm xy}(\omega',\omega)$ and $G_{{\rm xy},\Delta {\rm t}_1,{\rm k}}(\omega) = \int d\omega' G_{\Delta {\rm t}_1,{\rm k}}(\omega') G_{\rm xy}(\omega',\omega)$, respectively. Each term $(F_{\rm xy,k}(\omega)$ and $G_{{\rm xy},\Delta {\rm t}_1,{\rm k}}(\omega))$ indicates the output amplitude in the mode y for the given input amplitude $(F_{\rm k}(\omega)$ and $G_{\Delta {\rm t}_1,{\rm k}}(\omega))$ in the mode x via the BS-FWM effect.

Lastly, we consider a coincidence detection between the idler and signal modes for the final state $(|\psi_{\rm out,1}\rangle)$. For convenience, we rewrite the final state in equation (S12) as

$$|\psi_{\rm out}\rangle = \sum_{\rm k} r_{\rm k} \left(\int d\omega_s A_{\rm k}(\omega_{\rm s}) \hat{a}_{s}^{\dagger}(\omega_{\rm s}) + B_{\rm k}(\omega_{\rm s}) \hat{a}_{\rm i}^{\dagger}(\omega_{\rm s})\right) \times \left(\int d\omega_{\rm i} C_{\rm k}(\omega_{\rm i}) \hat{a}_{\rm s}^{\dagger}(\omega_{\rm i}) + D_{\rm k}(\omega_{\rm i}) \hat{a}_{\rm i}^{\dagger}(\omega_{\rm i})\right) |0\rangle\,. \tag{S13}$$

The two-photon detection of the final state at time t_1 in the signal mode and at time t_2 in the idler mode is described by

$$\hat{E}_{\rm s}(t_1)\hat{E}_{\rm i}(t_2)\,|\psi_{\rm out}\rangle = \sum_{\rm k} r_{\rm k} \int d\omega_1 d\omega_2 [A_{\rm k}(\omega_1) D_{\rm k}(\omega_2) + C_{\rm k}(\omega_1) B_{\rm k}(\omega_2)] \exp[-i\omega_1 t_1 - i\omega_2 t_2]\,|0\rangle\,, \tag{S14}$$

where the electric-field annihilation operator $E_{\rm x}(t)$ is $E_{\rm x}(t) = \int d\omega \hat{a}_x(\omega) e^{-i\omega t}$. The probability density of the two-photon detection is expressed as

$$\begin{aligned} ||\hat{E}_{\rm s}(t_1)\hat{E}_{\rm i}(t_2)\,|\psi_{\rm out}\rangle\,|^2 = \sum_{\rm k,\ k'} r_{\rm k} r_{\rm k'}^{*} \int d\omega_1' d\omega_2' d\omega_1 d\omega_2 [A_{\rm k'}^{*}(\omega_1') D_{\rm k'}^{*}(\omega_2') + C_{\rm k'}^{*}(\omega_1') B_{\rm k'}^{*}(\omega_2')] \\ \times [A_{\rm k}(\omega_1) D_{\rm k}(\omega_2) + C_{\rm k}(\omega_1) B_{\rm k}(\omega_2)] \exp[i(\omega_1' - \omega_1)t_1 + i(\omega_2' - \omega_2)t_2]. \end{aligned} \tag{S15}$$

The measured coincidence count is proportional to the time-averaged probability as the coherence time of the single photons ($\sim$ ps) is much finer than the temporal resolution of the single-photon detection (~ 100 ps). The time-averaged probability of the two-photon detection is given by

$$\begin{aligned} \int dt_1 dt_2 ||\hat{E}_{\rm s}(t_1)\hat{E}_{\rm i}(t_2)\,|\psi_{\rm out}\rangle\,|^2 = \sum_{\rm k,k'} r_{\rm k} r_{\rm k'}^{*} \int d\omega_1 d\omega_2 [A_{\rm k'}^{*}(\omega_1) D_{\rm k'}^{*}(\omega_2) + C_{\rm k'}^{*}(\omega_1) B_{\rm k'}^{*}(\omega_2)] \\ \times [A_{\rm k}(\omega_1) D_{\rm k}(\omega_2) + C_{\rm k}(\omega_1) B_{\rm k}(\omega_2)]. \end{aligned} \tag{S16}$$

By substituting the parameters in equation (S16) as $A_{\rm k}(\omega) = F_{\rm ss,k}(\omega)$, $B_{\rm k}(\omega) = F_{\rm si,k}(\omega)$, $C_{\rm k}(\omega) = G_{{\rm is},\Delta {\rm t}_1,{\rm k}}(\omega)$, and $D_{\rm k}(\omega) = G_{{\rm ii},\Delta {\rm t}_1,{\rm k}}(\omega)$, we can calculate the frequency-domain HOM effect.

Similarly, we are able to calculate the time-averaged probability of the two-photon detection either in the idler mode or the signal mode. The time-averaged probability in the signal mode is described as

$$\int dt_1 dt_2 ||\hat{E}_{\rm s}(t_1)\hat{E}_{\rm s}(t_2)\,|\psi_{\rm out}\rangle\,|^2 = \sum_{\rm k,k'} r_{\rm k} r^*_{\rm k'} \int d\omega_1 d\omega_2 [A^*_{\rm k'}(\omega_1)C^*_{\rm k'}(\omega_2) + C^*_{\rm k'}(\omega_1)A^*_{\rm k'}(\omega_2)] \times [A_{\rm k}(\omega_1)C_{\rm k}(\omega_2) + C_{\rm k}(\omega_1)A_{\rm k}(\omega_2)], \tag{S17}$$

and the time-averaged probability in the idler mode is represented as

$$\int dt_1 dt_2 ||\hat{E}_{\rm i}(t_1)\hat{E}_{\rm i}(t_2)\,|\psi_{\rm out}\rangle\,|^2 = \sum_{\rm k,k'} r_{\rm k} r^*_{\rm k'} \int d\omega_1 d\omega_2 [B^*_{\rm k'}(\omega_1)D^*_{\rm k'}(\omega_2) + D^*_{\rm k'}(\omega_1)B^*_{\rm k'}(\omega_2)] \times [B_{\rm k}(\omega_1)D_{\rm k}(\omega_2) + D_{\rm k}(\omega_1)B_{\rm k}(\omega_2)]. \tag{S18}$$

Fig. S3 represents the simulated frequency-domain HOM patterns for various BS-FWM pump powers and optical fiber lengths. Fig. S3a is calculated with the joint spectral intensity from Fig. S2a where the fiber length is set to 200 m. The simulated HOM-dip visibilities are 90.5%, 95.6%, and 92.9% for the BS-FWM pump powers of 4.74 W, 5.20 W, and 5.76 W, respectively. Fig. S3b is based on the joint spectral intensity from Fig. S2b, where the fiber length is set to 50 m. The simulated HOM-dip visibilities are 93.7%, 99.0%, and 96.2% for the BS-FWM pump powers of 4.74 W, 5.20 W, and 5.76 W, respectively. As seen from Fig. S3, the joint-spectral intensity of the 50-m optical fiber shows a more symmetric pattern compared to its 200-m counterpart. This symmetry elevates the visibilities, as a result of the more balanced interference.

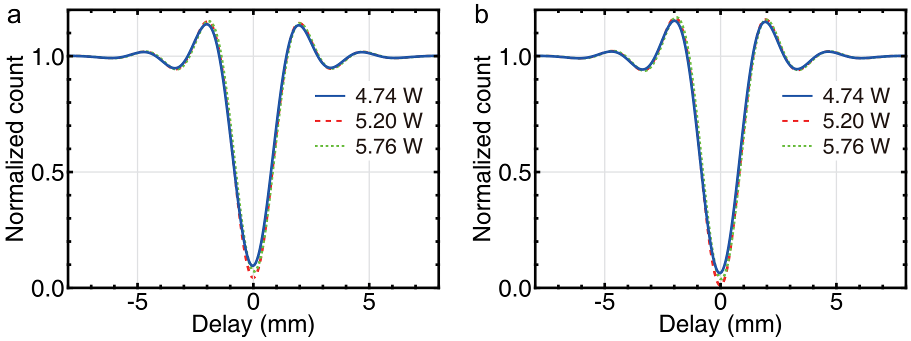

Figure S3. **Simulated frequency-domain HOM effect.** **a**, HOM-dip patterns corresponding to various BS-FWM pump powers, where the length of the optical fiber is set to 200 m. **b**, HOM-dip patterns when the length of the optical fiber is set to 50 m. The BS-FWM pump powers for the blue solid, red dashed, and green dotted curves are 4.74 W, 5.20 W, and 5.76 W, respectively.

B. Frequency-domain NOON-state interference

The NOON-state interference is experimentally implemented via three steps. First, the NOON state is prepared with the frequency-domain HOM effect via the BS-FWM effect. Then, the relative phase between the NOON state is introduced by the delay line (DL2). Finally, the frequency modes of the NOON state are mixed by another BS-FWM effect, leading to the observation of the NOON-state interference. Starting from the state in equation (S12), which describes the HOM effect in the frequency domain, the introduced delay Δt_2 transforms the state as

$$|\psi_{\rm out,2}\rangle = \sum_{\rm k} r_{\rm k} \left(\int d\omega_{\rm s} T(\omega_{\rm s},\omega_{\rm s0},\Delta t_2) F_{\rm ss,k}(\omega_{\rm s})\hat{a}^\dagger_{\rm s}(\omega_{\rm s}) + T(\omega_{\rm s},\omega_{\rm i0},\Delta t_2) F_{\rm si,k}(\omega_{\rm s})\hat{a}^\dagger_{\rm i}(\omega_{\rm s}) \right) \times \left(\int d\omega_{\rm i} T(\omega_{\rm i},\omega_{\rm s0},\Delta t_2) G_{\rm is,\Delta t_1,k}(\omega_{\rm i})\hat{a}^\dagger_{\rm s}(\omega_{\rm i}) + T(\omega_{\rm i},\omega_{\rm i0},\Delta t_2) G_{\rm ii,\Delta t_1,k}(\omega_{\rm i})\hat{a}^\dagger_{\rm i}(\omega_{\rm i}) \right) |0\rangle\,, \tag{S19}$$

where the temporal delay $T(\omega_{\rm x},\omega_{\rm y0},\Delta t_2) = \exp[i(\omega_{\rm x}+\omega_{\rm y0})\Delta t_2]$. The effect of the other BS-FWM is described as

$$
\begin{aligned}
|\psi_{\mathrm{out},3}\rangle &= \sum_{\mathrm{k}} r_{\mathrm{k}} \left(\int d\omega_1 A'_{\mathrm{k}}(\omega_1)\hat{a}^{\dagger}_{\mathrm{s}}(\omega_1) + B'_{\mathrm{k}}(\omega_1)\hat{a}^{\dagger}_{\mathrm{i}}(\omega_1) \right) \times \left(\int d\omega_2 C'_{\mathrm{k}}(\omega_2)\hat{a}^{\dagger}_{\mathrm{s}}(\omega_2) + D'_{\mathrm{k}}(\omega_2)\hat{a}^{\dagger}_{\mathrm{i}}(\omega_2) \right) |0\rangle \,, \\
A'_{\mathrm{k}}(\omega_1) &= \int d\omega_{\mathrm{s}} G'_{\mathrm{ss}}(\omega_{\mathrm{s}},\omega_1) T(\omega_{\mathrm{s}},\omega_{\mathrm{s0}},\Delta t_2) F_{\mathrm{ss,k}}(\omega_{\mathrm{s}}) + G'_{\mathrm{is}}(\omega_{\mathrm{s}},\omega_1) T(\omega_{\mathrm{s}},\omega_{\mathrm{i0}},\Delta t_2) F_{\mathrm{si,k}}(\omega_{\mathrm{s}}), \\
B'_{\mathrm{k}}(\omega_1) &= \int d\omega_{\mathrm{s}} G'_{\mathrm{si}}(\omega_{\mathrm{s}},\omega_1) T(\omega_{\mathrm{s}},\omega_{\mathrm{s0}},\Delta t_2) F_{\mathrm{ss,k}}(\omega_{\mathrm{s}}) + G'_{\mathrm{ii}}(\omega_{\mathrm{s}},\omega_1) T(\omega_{\mathrm{s}},\omega_{\mathrm{i0}},\Delta t_2) F_{\mathrm{si,k}}(\omega_{\mathrm{s}}), \\
C'_{\mathrm{k}}(\omega_2) &= \int d\omega_{\mathrm{i}} G'_{\mathrm{ss}}(\omega_{\mathrm{i}},\omega_2) T(\omega_{\mathrm{i}},\omega_{\mathrm{s0}},\Delta t_2) G_{\mathrm{is},\Delta t_1,\mathrm{k}}(\omega_{\mathrm{i}}) + G'_{\mathrm{is}}(\omega_{\mathrm{i}},\omega_2) T(\omega_{\mathrm{i}},\omega_{\mathrm{i0}},\Delta t_2) G_{\mathrm{ii},\Delta t_1,\mathrm{k}}(\omega_{\mathrm{i}}), \\
D'_{\mathrm{k}}(\omega_2) &= \int d\omega_{\mathrm{i}} G'_{\mathrm{si}}(\omega_{\mathrm{i}},\omega_2) T(\omega_{\mathrm{i}},\omega_{\mathrm{s0}},\Delta t_2) G_{\mathrm{is},\Delta t_1,\mathrm{k}}(\omega_{\mathrm{i}}) + G'_{\mathrm{ii}}(\omega_{\mathrm{i}},\omega_2) T(\omega_{\mathrm{i}},\omega_{\mathrm{i0}},\Delta t_2) G_{\mathrm{ii},\Delta t_1,\mathrm{k}}(\omega_{\mathrm{i}}).
\end{aligned}
\tag{S20}
$$

Note that $G'_{\mathrm{xy}}(\omega_1,\omega_2)$ is the different Green function from $G_{\mathrm{xy}}(\omega_1,\omega_2)$ as the power of the BS-FWM pump for the second stage may be different. By substituting equations (S17, S18) with the parameters $A(\omega) = A'(\omega)$, $B(\omega) = B'(\omega)$, $C(\omega) = C'(\omega)$, and $D(\omega) = D'(\omega)$, we theoretically calculate the NOON-state interference in the frequency domain.

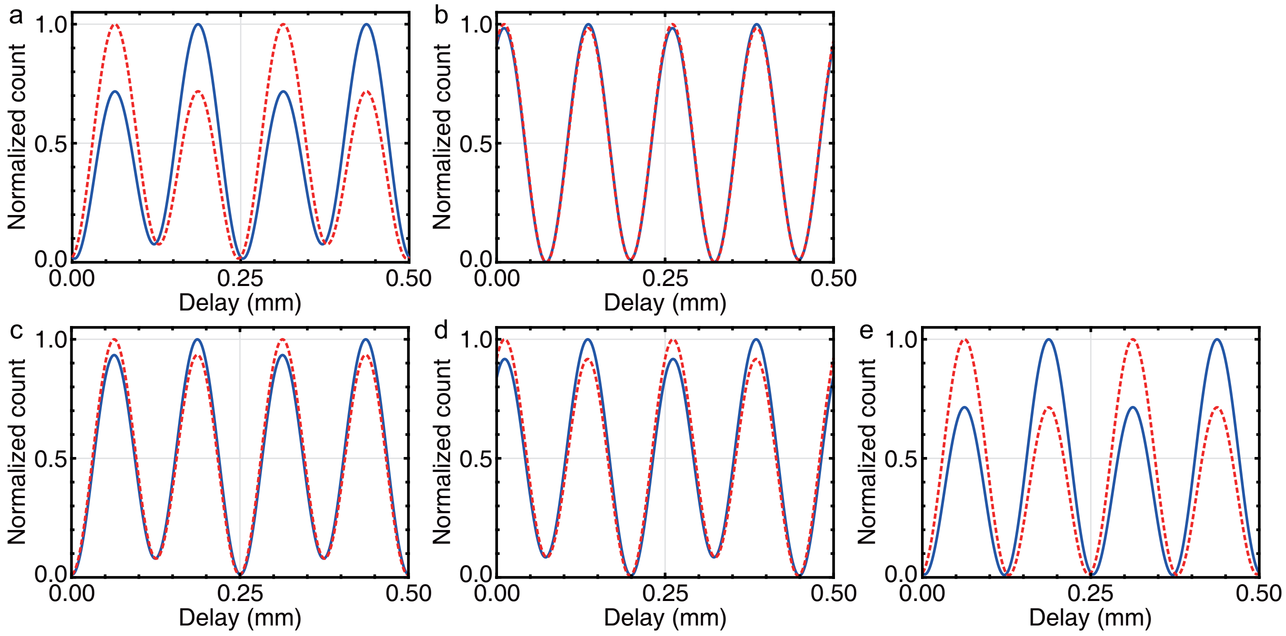

Figure S4. **Simulated frequency-domain NOON-state interference.** NOON-state interference patterns under various conditions. **a**, The experimental condition. **b**, Mitigating the three effects. **c**, Reducing asymmetry in the two-photon state. **d**, Compensating walk-off between the two photons. **e**, Balancing BS-FWM pump powers.

Fig. S4 illustrates the frequency-domain NOON-state interference patterns across various simulated conditions. The blue solid curve and red dashed curve denote the normalized counts for the signal and idler modes, respectively. Fig. S4a presents the interference patterns under our experimental condition. We identify three primary contributors to the observed non-sinusoidal patterns and non-ideal visibilities: 1) asymmetry in the generated two-photon state, 2) walk-off between the two photons in the NZDSF, 3) unbalanced BS-FWM pump powers, 5.76 W for the first stage and 4.74 W for the second. Fig. S4b represents the interference patterns after rectifying the above three effects. Here, the fitted visibilities of the fringes are 99.1% for the signal mode and 99.0% for the idler mode. The three effects are mitigated by the following procedures: 1) Reducing the length of the single-mode fiber from 200 m to 50 m, thereby achieving a symmetrical two-photon state as seen from Fig. S2, 2) Introducing a 3.23 ps delay to the idler photon after the first-stage BS-FWM process, 3) Equalizing BS-FWM pump powers to 5.20 W for both stages. Fig. S4(c-e) presents the interference patterns while individually addressing the effects: c) Compensating for the two-photon state asymmetry, d) Canceling the walk-off effect, e) Balancing the BS-FWM pump powers. Therefore, we successfully simulate the frequency-domain NOON-state interference patterns and explain the non-sinusoidal patterns and non-ideal visibilities. The three influential factors for the non-ideal patterns are the asymmetry in the generated two-photon state, the walk-off effect, and the unbalanced BS-FWM pump powers.

C. Frequency-domain single-photon inteference

The single-photon interference is implemented by heralding the idler photon while the signal photon evolves in the interferometer. As the signal and idler photons propagate in the different path modes, equation (S9) is modified as

$$|\psi_{\mathrm{in},2}\rangle = \sum_{\mathrm{k}} r_{\mathrm{k}} \int d\omega_{\mathrm{s}} F_{\mathrm{k}}(\omega_{\mathrm{s}}) \hat{a}^{\dagger}_{\mathrm{s1}}(\omega_{\mathrm{s}}) \int d\omega_{\mathrm{i}} G_{\mathrm{k}}(\omega_{\mathrm{i}}) \hat{a}^{\dagger}_{\mathrm{i2}}(\omega_{\mathrm{i}}) |0\rangle , \tag{S21}$$

where $\hat{a}^{\dagger}_{\mathrm{s1}}$ indicates the photon creation operator at the signal frequency mode with the path mode 1, and $\hat{a}^{\dagger}_{\mathrm{i2}}$ is the creation operator at the idler frequency mode with the path mode 2. After the signal photon evolves in the interferometer, the final state is expressed as

$$|\psi_{\mathrm{out},4}\rangle = \sum_{\mathrm{k}} r_{\mathrm{k}} \left(\int d\omega_1 A'_{\mathrm{k}}(\omega_1) \hat{a}^{\dagger}_{\mathrm{s1}}(\omega_1) + B'_{\mathrm{k}}(\omega_1) \hat{a}^{\dagger}_{\mathrm{i1}}(\omega_1) \right) \times \int d\omega_2 G_{\mathrm{k}}(\omega_2) \hat{a}^{\dagger}_{\mathrm{i2}}(\omega_2) |0\rangle . \tag{S22}$$

where $A'_{\mathrm{k}}(\omega)$ and $B'_{\mathrm{k}}(\omega)$ are equivalent with Equation (S20). The two-photon detection of the signal photon at the path mode 1 and time t_1, and the idler photon at the path mode 2 and time t_2, is represented as

$$\hat{E}_{\mathrm{s1}}(t_1) \hat{E}_{\mathrm{i2}}(t_2) |\psi_{\mathrm{out},4}\rangle = \sum_{\mathrm{k}} r_{\mathrm{k}} \int d\omega_1 \exp[-i\omega_1 t_1] A'_{\mathrm{k}}(\omega_1) \int d\omega_2 \exp[-i\omega_2 t_2] G_{\mathrm{k}}(\omega_2) |0\rangle , \tag{S23}$$

and the probability density of the two-photon detection is calculated as follows,

$$||\hat{E}_{\mathrm{s1}}(t_1) \hat{E}_{\mathrm{i2}}(t_2) |\psi_{\mathrm{out},4}\rangle|^2 = \sum_{\mathrm{k,k'}} r_{\mathrm{k}} r_{\mathrm{k'}} \int d\omega'_1 d\omega'_2 d\omega_1 d\omega_2 A'^{*}_{\mathrm{k'}}(\omega'_1) G^{*}_{\mathrm{k'}}(\omega'_2) A'_{\mathrm{k}}(\omega_1) G_{\mathrm{k}}(\omega_2) \exp[i(\omega_1 - \omega'_1)t_1 + i(\omega_2 - \omega'_2)t_2]. \tag{S24}$$

The time-averaged probability, which is proportional to the measured coincidence count, of the photons at the signal mode at path mode 1 and the idler mode at path mode 2 is calculated as

$$\int dt_1 dt_2 ||\hat{E}_{\mathrm{s1}}(t_1) \hat{E}_{\mathrm{i2}}(t_2) |\psi_{\mathrm{out},4}\rangle|^2 = \sum_{\mathrm{k,k'}} r_{\mathrm{k}} r_{\mathrm{k'}} \int d\omega_1 A'^{*}_{\mathrm{k'}}(\omega_1) A'_{k}(\omega_1) \int d\omega_2 G^{*}_{\mathrm{k'}}(\omega_2) G_{\mathrm{k}}(\omega_2) = \sum_{\mathrm{k}} r^2_{\mathrm{k}} \int d\omega_1 |A'_{\mathrm{k}}(\omega_1)|^2, \tag{S25}$$

where the orthogonality between the Schmidt modes leads to a relation, $\int d\omega G^{*}_{\mathrm{k'}}(\omega) G_{\mathrm{k}}(\omega) = \delta_{\mathrm{k,k'}}$. With the same procedures, the time-averaged probability of the idler mode at the path mode 1 and the idler mode at the path mode 2 is calculated as follows,

$$\int dt_1 dt_2 ||\hat{E}_{\mathrm{i1}}(t_1) \hat{E}_{\mathrm{i2}}(t_2) |\psi_{\mathrm{out},4}\rangle|^2 = \sum_{\mathrm{k,k'}} r_{\mathrm{k}} r_{\mathrm{k'}} \int d\omega_1 B'^{*}_{\mathrm{k'}}(\omega_1) B'_{k}(\omega_1) \int d\omega_2 G^{*}_{\mathrm{k'}}(\omega_2) G_{\mathrm{k}}(\omega_2) = \sum_{\mathrm{k}} r^2_{\mathrm{k}} \int d\omega_1 |B'_{\mathrm{k}}(\omega_1)|^2. \tag{S26}$$

Fig. S5 depicts the frequency-domain single-photon interference patterns under various simulated conditions. The blue solid curve and red dashed curve denote the normalized counts for the signal and idler modes, respectively. Fig. S5a presents the interference patterns under our experimental condition. Similar to the previous discussion, three primary factors contribute to the observed non-ideal visibilities: 1) asymmetry in the single-photon spectrum (signal photon), 2) walk-off between the two photons within the NZDSF, 3) Unbalanced BS-FWM pump powers, 5.76 W for the first stage and 4.74 W for the second. Notably, the asymmetry in the two-photon state leads to asymmetry in the single-photon spectrum. Fig. S5b presents the interference patterns after mitigating the above three effects, where the fitted visibilities of the fringes are 98.1% for the signal mode and 98.6% for the idler modes. The three effects are rectified by the following methods: 1) Reducing the length of the single-mode fiber from 200 m to 50 m, which symmetrize the single-photon spectrum. 2) Introducing a 3.23 ps delay to the idler photon after the first-stage BS-FWM process, 3) Balancing BS-FWM pump powers to 5.20 W for both stages. Fig. S5(c-e) presents the interference patterns by individually addressing the effects: c) Reducing the asymmetry in the single-photon spectrum, d) Compensating the walk-off effect, e) Balancing the BS-FWM pump powers. Therefore, we simulate the frequency-domain single-photon interference patterns and explain the non-ideal visibilities. The three influential factors for the non-ideal visibilities are the asymmetry in the single-photon spectrum, the walk-off effect, and the unbalanced BS-FWM pump powers.

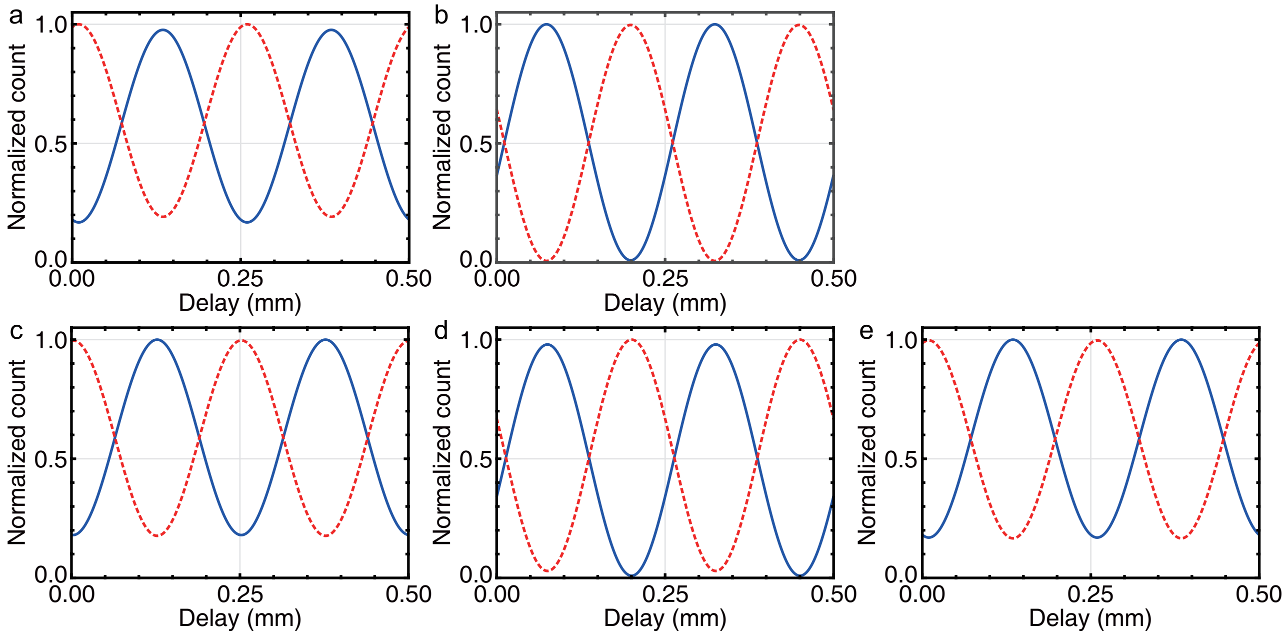

Figure S5. **Simulated frequency-domain single-photon interference.** Single-photon interference patterns under various conditions. **a**, The experimental condition. **b**, Mitigating the three effects. **c**, Reducing asymmetry in the two-photon state. **d**, Compensating walk-off between the two photons. **e**, Balancing BS-FWM pump powers.

IV. FREQUENCY-DOMAIN HOM INTERFERENCE WITHOUT ACCIDENTAL SUBTRACTION

In Fig. S6, we illustrate the visibilities of the frequency-domain HOM interference as a function of the input BS-FWM power, which determines the splitting ratio of the frequency beam splitter. The blue squares and the red diamonds represent the raw visibilities for the forward and backward directions of the BS-FWM processes, respectively. The error bars are derived from the fitting coefficients and confidence bounds of the measured HOM dips, where the fitting function is denoted as a Gaussian function multiplied by a sinc function. The blue solid and red dashed curves depict the simulated HOM visibilities for the forward and backward directions, respectively. Although the experimental result without the accidental subtraction shows a discrepancy with the simulation result, the overall trends are well consistent.

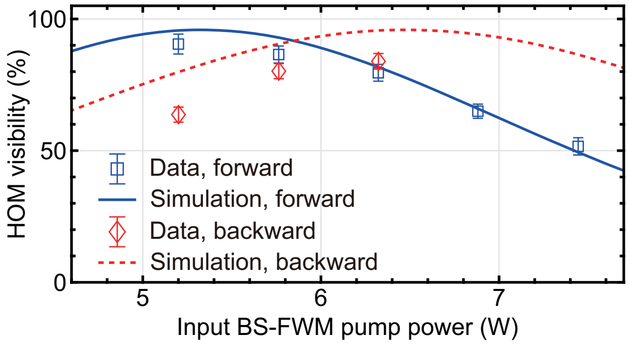

Figure S6. **Frequency-domain HOM interference without accidental subtraction.** Raw visibility of the HOM interference against the input BS-FWM power. The blue squares and red diamonds represent the raw visibilities of the HOM effect for the forward and backward directions, respectively, and the blue solid and red dashed curves indicate the corresponding simulation results.

V. FISHER INFORMATION

While we observe super-resolution, a two-fold enhancement compared to single-photon interference in Figure 5, achieving super-sensitivity remains elusive due to high optical loss in our experimental setup.

In the quantum estimation theory, the lower bound of phase uncertainty, $\Delta\phi_{\mathrm{low}}$, is linked to quantum Fisher information, $F_{\mathrm{Q}}(\phi)$, by the relation $\Delta\phi_{\mathrm{low}} = 1/\sqrt{qF_{\mathrm{Q}}(\phi)}$, where '$q$' is the number of repeated measurements[12,13]. For the NOON state, quantum Fisher information is given by $F_{\mathrm{Q}}(\phi) = N^2V^2\eta_{\mathrm{sys,coincidence}}\eta_{\mathrm{g}}$, where V is the visibility of the interference fringe, $\eta_{\mathrm{sys,coincidence}}$ is the system efficiency, and η_{g} is the generation efficiency of the NOON state[14]. Under our experimental conditions, the visibility, system efficiency, and generation efficiency are measured at 67%, 0.0039%, and 100%, respectively. This leads to a calculated quantum Fisher information of 7.0×10^{-5}, resulting in a lower bound of $\Delta\phi_{\mathrm{low}} = 120$ for a single-shot measurement ($q = 1$). Since the lower bound exceeds the phase uncertainty of the single-photon interference, $\Delta\phi_{\mathrm{low,single}} = 1/(V\sqrt{\eta_{\mathrm{sys,single}}\eta_{\mathrm{g}}}) = 13$, achieving supersensitivity is currently unattainable. In the single-photon interference experiment, the visibility, system efficiency ($\eta_{\mathrm{sys,single}}$), and generation efficiency are 70%, 1.2%, and 100%, respectively.

We expect that the high optical loss can be significantly improved through customizing optical components or implementing the experimental setup on a single chip, such as Silicon or Silicon-nitride platforms. However, we believe that this is beyond the scope of our current paper.

VI. FREQUENCY-DOMAIN NOON-STATE INTERFERENCE WITHOUT ACCIDENTAL SUBTRACTION

Fig. S7 represents the frequency-domain NOON-state interference and single-photon interference without accidental subtraction. Fig. S7a depicts the NOON-state interference. Blue squares and red triangles, accompanied by error bars, indicate measured coincidence counts between the SNSPDs D1 & D2 and D3 & D4, respectively. In contrast, markers without error bars present accidental coincidence counts. Each data point is measured for 60 seconds. Solid blue and dashed red curves are the simulation results, and the error bars are calculated assuming Poissonian statistics of the detection. Fig. S7b represents the single-photon interference. Blue squares and red triangles, with error bars, indicate measured coincidence counts between the SNSPDs D1 & D5 and D3 & D5, respectively. Markers without error bars present accidental coincidence counts. Each point is measured for a second. The experimental and simulation results are well consistent.

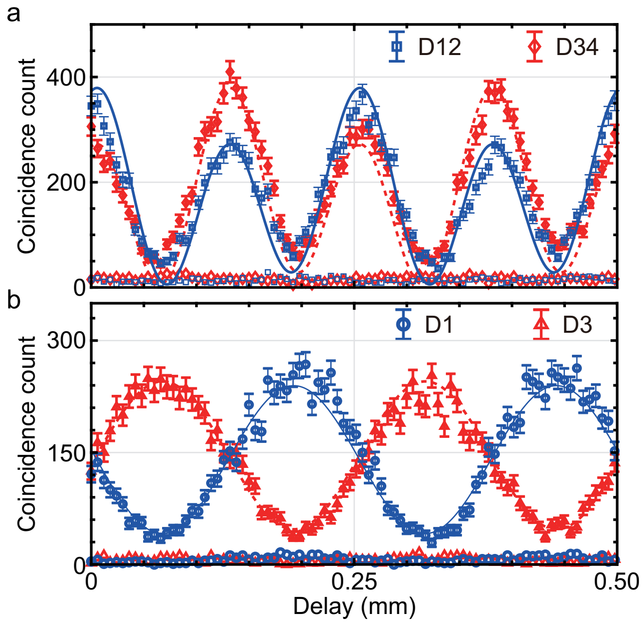

Figure S7. **Frequency-domain NOON-state interference without accidental subtraction. a**, Coincidence counts against the delay (DL2) for the NOON-state interference (N = 2). Blue squares and red diamonds with error bars represent coincidence counts between the SNSPDs D1 & D2 and D3 & D4, respectively, where markers without error bars indicate the accidental coincidence counts. Solid blue and dashed red curves are simulation results. The error bars are calculated assuming Poissonian statistics of the detection. **b**, Coincidence counts against the delay (DL2) for the single-photon interference. Blue circle and red triangles with error bars indicate the coincidence counts between the SNSPDs D1 & D5 and D3 & D5, respectively, where markers without error bars depict the accidental coincidence counts.

[1] McKinstrie, C., Harvey, J., Radic, S. & Raymer, M. Translation of quantum states by four-wave mixing in fibers. *Optics Express* **13**, 9131–9142 (2005).

[2] McGuinness, H. J., Raymer, M. G., McKinstrie, C. J. & Radic, S. Quantum frequency translation of single-photon states in a photonic crystal fiber. *Physical review letters* **105**, 093604 (2010).

[3] Clemmen, S., Farsi, A., Ramelow, S. & Gaeta, A. L. Ramsey interference with single photons. *Physical review letters* **117**, 223601 (2016).

[4] Kobayashi, T. *et al.* Frequency-domain hong–ou–mandel interference. *Nature photonics* **10**, 441–444 (2016).

[5] Joshi, C. *et al.* Frequency-domain quantum interference with correlated photons from an integrated microresonator. *Physical review letters* **124**, 143601 (2020).

[6] Clark, A. S., Shahnia, S., Collins, M. J., Xiong, C. & Eggleton, B. J. High-efficiency frequency conversion in the single-photon regime. *Optics letters* **38**, 947–949 (2013).

[7] Park, K., Lee, D., Boyd, R. W. & Shin, H. Telecom c-band photon-pair generation using standard smf-28 fiber. *Optics Communications* **484**, 126692 (2021).

[8] McGuinness, H., Raymer, M. & McKinstrie, C. Theory of quantum frequency translation of light in optical fiber: application to interference of two photons of different color. *Optics express* **19**, 17876–17907 (2011).

[9] Dudley, J. M. & Taylor, J. R. *Supercontinuum generation in optical fibers* (Cambridge University Press, 2010).

[10] Agrawal, G. Chapter 2 - pulse propagation in fibers. In Agrawal, G. (ed.) *Nonlinear Fiber Optics (Fifth Edition)*, Optics and Photonics, 27–56 (Academic Press, Boston, 2013), fifth edition edn. URL https://www.sciencedirect.com/science/article/pii/B9780123970237000024.

[11] Lee, D., Park, K., Shin, W. & Shin, H. Translation from a distinguishable to indistinguishable two-photon state. *ACS Photonics* **10**, 3359–3365 (2023).

[12] Braunstein, S. L. & Caves, C. M. Statistical distance and the geometry of quantum states. *Physical Review Letters* **72**, 3439 (1994).

[13] Fisher, R. A. Theory of statistical estimation. In *Mathematical proceedings of the Cambridge philosophical society*, vol. 22, 700–725 (Cambridge University Press, 1925).

[14] Shin, H., Magaña-Loaiza, O. S., Malik, M., O'Sullivan, M. N. & Boyd, R. W. Enhancing entangled-state phase estimation by combining classical and quantum protocols. *Optics Express* **21**, 2816–2822 (2013).
